# *Clostridium perfringens* Type E Virulence Traits Involved in Gut Colonization

**DOI:** 10.1371/journal.pone.0121305

**Published:** 2015-03-23

**Authors:** Leandro M. Redondo, Juan M. Díaz Carrasco, Enzo A. Redondo, Fernando Delgado, Mariano E. Fernández Miyakawa

**Affiliations:** 1 Instituto de Patobiología, Centro Nacional de Investigaciones Agropecuarias, Instituto Nacional de Tecnología Agropecuaria, Castelar, Buenos Aires, Argentina; 2 Consejo Nacional de Investigaciones Científicas y Técnicas, Ciudad Autónoma de Buenos Aires, Argentina; Institute Pasteur, FRANCE

## Abstract

*Clostridium perfringens* type E disease in ruminants has been characterized by hemorrhagic enteritis or sudden death. Although type E isolates are defined by the production of alpha and iota toxin, little is known about the pathogenesis of *C*. *perfringens* type E infections. Thus far, the role of iota toxin as a virulence factor is unknown. In this report, iota toxin showed positive effects on adherence and colonization of *C*. *perfringens* type E while having negative effect on the adherence of type A cells. *In-vitro* and *in-vivo* models suggest that toxinotype E would be particularly adapted to exploit the changes induced by iota toxin in the surface of epithelial cells. In addition, type E strains produce metabolites that affected the growth of potential intra-specific competitors. These results suggest that the alteration of the enterocyte morphology induced by iota toxin concomitantly with the specific increase of type E cell adhesion and the strong intra-specific growth inhibition of other strains could be competitive traits inherent to type E isolates that improve its fitness within the bovine gut environment.

## Introduction

*Clostridium perfringens*, a Gram positive, anaerobic and spore forming bacteria, is widely distributed in nature and considered an important pathogen of both human and livestock [1]. Isolates are classified into five toxinotypes (A to E) based upon production of four major toxins (alpha, beta, epsilon, and iota) [2]. *C*. *perfringens* type E isolates are defined by the production of alpha and iota toxin, although additional potential virulence genes encoding beta2-toxin, urease or lambda-toxin can be also found in some isolates [3]. Type E isolates cause enteritis or enterotoxaemia in rabbits [4], lambs [2], cattle [5, 6, 7], goats [8] and dogs [1]. *C*. *perfringens* type E disease in ruminants is characterized by hemorrhagic enteritis or sudden death and has been described in ovine and bovines. Lesions observed at necropsy are hyperemia and edema in the mucosa of intestine and abomasum, with foci of hemorrhage, acute inflammation and submucosal edema [6, 7]. Since first description more than 50 years ago [8], toxinotype E has been considered an uncommon cause of enteritis in calves [9] although this toxinotype could be more common than previously considered [6, 7, 10].

Information available about type E pathogenesis is scarce, but it is generally accepted that iota toxin (ITX) is the main virulence factor of *C*. *perfringens* type E isolates [6, 11]. ITX belongs to the binary toxin family which is composed of two unlinked protein, an enzymatic component with ADP-ribosyltransferase activity and a binding component which binds to the cell surface receptor [12, 13] and facilitates the enzymatic component entry into the cytosol [14, 15]. Besides ITX, other binary toxins are codified by *Clostridium spp*. strains that produce intestinal diseases as diarrhea and enteritis including *C*. *botulinum* C2 toxin [16], *C*. *spiroforme* iota-like toxin [17] and *C*. *difficile* ADP-ribosyltransferase [18]. Cellular intoxication by binary toxins has been extensively studied and most recent reports suggest that actin depolymerization induced by *C*. *difficile´s* binary toxin triggers the formation of microtubule-based protrusions at the apical side of cells, and reroutes secretory vesicles containing extracellular matrix proteins like fibronectin from basolateral to the apical surface of host cells. Eventually, secreted fibronectin and the microtubule-based meshwork increase the adherence of clostridia [19, 20]. Currently, the amount of information regarding the ability of *C*. *perfringens* strains to adhere to epithelial cells is limited [21, 22].

In natural cases of hemorrhagic enteritis produced by *C*. *perfringens* type E, a clonal predominance among isolates from affected animals has been observed [7, 23]. In contrast, a high degree of genetic diversity is usually found in *C*. *perfringens* strains obtained from healthy animals. These observations together with the multilocus sequence typing (MLST) analysis of type E isolates from diverse geographical regions suggest that toxinotype E has specialized to succeed in a particular ecological niche, such as the bovine gut. Also, recent evidence shows that some *C*. *perfringens* are able to produce bacteriocins to inhibit other *C*. *perfringens* strains growth [24, 25], suggesting that intra-specific inhibition could be an important mechanism involved in the clonal predominance observed in *C*. *perfringens* type E enteritis.

In this work, we observed that *C*. *perfringens* type E strains produce metabolites other than ITX which affect the growth of potential competitors, while ITX induces modifications on epithelial cells that increase the adherence of type E strains while having negative effect on the adherence of other *C*. *perfringens* strains. Both effects seem to be important to increase the relative fitness of type E strains within the intestinal environment.

## Materials and Methods

### Bacterial strains and culture conditions

Two field isolates of *C*. *perfringens* type E (CpE218 and CpE132) obtained during two outbreaks of hemorrhagic enteritis in cattle [7], and two type A strains isolated from healthy cattle (Cp31 and Cp88) were used in the present study. Additional information about the strains is presented in Table 1. *C*. *perfringens* isolates were plated in blood agar plates directly from the freezer stock and incubated overnight at 37°C under anaerobic conditions. For the purposes of this study, single colonies were cultured in 10 ml of BHI broth in individual tubes overnight at 37°C in anaerobic chambers. To choose the most effective selective media for subsequent tests, bacterial cultures were then spread on individual blood agar plates containing neomycin (100 μg/ml) [26] with or without nalidixic acid (25 μg/ml). The identity of the colonies was confirmed by biochemical tests and multiplex PCR [27].

**Table 1:** Strains used in this study.

**Strain**	**Type**	**Genotype**	**Host specie**	**Origin**
Cp31	A	*cpa*	Bovine	Healthy
Cp88	A	*cpa*	Bovine	Healthy
Cp132	E	*cpa*, *cpe*, *cpb2*, *iA*	Bovine	Hemorraghic enteritis
CpE218	E	*cpa*, *cpe*, *cpb2*, *iA*	Bovine	Hemorraghic enteritis

### Growth curves

0.1 ml of overnight cultures prepared as mentioned above was used to inoculate 10 ml of fresh BHI and incubated at 37°C under anaerobic conditions. Aliquots were taken every 2 h and the optical density was measured at 600 nm. The final value was taken as the average of three independent measurements [28].

### Caco-2 cell culture

Human-derived enterocyte-like Caco-2 cells were cultured in Eagle’s MEM supplemented with heat-inactivated 10% fetal calf serum, 1% non-essential amino acids, 1% glutamine, penicillin-streptomycin (100 IU/ml and 100 μg/ml respectively) in a humidified atmosphere containing 5% CO_2_ at 37°C. For adhesion assays, the Caco-2 cells were cultured until confluence and then further cultivated for 7–10 days to obtain differentiated cells [22]. Caco-2 cells medium was replaced with antibiotic-free medium for 24 h to perform the adhesion assay.

### Agar spot on lawn assays

This method was used for screening inhibitory activity between *C*. *perfringens* isolates. Selected strains were cultured until the late stationary phase and diluted with fresh BHI to reach a density of 0.5 in McFarland scale. Bacterial suspension was spread on a BHI agar plate to form a homogeneous lawn. Another aliquot of the same culture was centrifuged at 13,000 g for 10 min to pull down bacteria and supernatants were collected and filter sterilized with 0.45 μm filters. Inoculated plates were allowed to dry for a few minutes and a drop of the filtered supernatants was placed over. Inhibitory abilities were tested in a chessboard fashion after incubation at 37°C in anaerobic atmosphere [25].

### Characterization of the antimicrobial soluble factor

Ammonium sulphate-concentrated *C*. *perfringens* Type E cell-free supernatant was used to test thermal and protease stability of the antimicrobial soluble factor. The remaining antibacterial activity after heat and protease treatment was determined by the agar spot test using dilutions of the treated supernatant and compared with the activity of the corresponding control (= 100%). Thermal stability was evaluated by determination of the residual antibacterial activity after incubation at 4, 24, 37, 42, 60, 80 and 100°C for 10, 30 and 60 min. Effect of trypsin, papain and proteinase K on antimicrobial activity was also tested. Each enzyme was prepared at a concentration of 10 mg/ml and added to the concentrated supernatant at a final concentration of 1 mg/ml. After incubation for 1 h at 37°C, the inhibitory activity was determined.

### Inhibition assays in liquid medium

Overnight cultures of *C*. *perfringens* type E (CpE218 strain) and *C*. *perfringens* type A (Cp88 strain) were diluted and inoculated into 10 ml of BHI broth (inhibitor/target ratio 1:10; that is, type E bacteria [1x10^6^ CFU/ml] to type A bacteria [1x10^7^ CFU/ml]). The same amount of type A bacteria was individually inoculated into 10 ml of BHI broth as control (monoculture). Another co-culture with a nalidixic acid sensitive type A strain (Cp31), which did not show inhibitory effect over the Cp88 strain, was prepared identically as Cp88+CpE218 mix and included as an additional control. All three cultures were incubated under anaerobic conditions at 37°C for 24 h and samples were collected at different time points (2, 4, 6 and 24 h). Samples were serially diluted and placed on blood agar plates supplemented with neomycin (100 mg/L) and nalidixic acid (25 mg/L). Blood agar plates supplemented with neomycin and nalidixic acid only allow Cp88 strain (type A) growth. Therefore this selective media was used for nalidixic acid resistant CFU counts in further described co-culture assays. Plates were cultured overnight at 37°C under anaerobic conditions. Colonies in each of the plates were counted and expressed as CFU/ml. Also at mentioned time intervals, OD600 of each culture was determined to evaluate total clostridia. Identity of the colonies and selective media specificity were confirmed by multiplex PCR [27].

### Competitive exclusion and strain displacement in a cell model

For competitive exclusion assays, Caco-2 cells monolayers were infected with a bacterial suspension. One ml of a stationary phase culture of each strain was centrifuged at 13.000 g for 5 min and washed 3 times with sterile PBS. Pellet was re-suspended in fresh MEM to get a final concentration of 1x10^6^ CFU of type E strain (CpE 218) and 1x10^7^ CFU of type A strain (Cp88) (inhibitor/target ratio 1:10). For competitive exclusion assay both strains were added simultaneously and incubated for 2 h in aerobic atmosphere. For displacement assay, type A bacteria suspension was added first to cell monolayer and allowed to attach for 2 h, washed 3 times with PBS to remove unattached bacteria and then type E bacteria suspension was added and incubated for another hour. After incubation, in both experiments, each well was washed 3 times with sterile PBS to remove no adhered bacteria. Sterile distilled water was added for 15 minutes to lyse the Caco-2 cells and recover attached bacteria. Finally, *C*. *perfringens* nalidixic acid resistant CFU number was determined as mentioned above.

### Iota toxin

ITX was purified as described by Stiles [29]. Briefly, cell-free supernatant proteins were precipitated by adding a saturated solution of ammonium sulphate (4°C) up to 70% saturation. Precipitate was collected 18 h later by centrifugation (7,000 g for 20 min), dissolved in 10 mM Tris-HCl buffer, pH 7.5 and dialyzed with the same buffer to remove residual ammonium sulphate. For ITX purification, concentrated supernatant was applied to a DEAE-Sepharose CL-6B column previously equilibrated with 10 mM Tris-HCl buffer, pH 7.5. Both fractions of ITX were eluted with a 0.0 to 0.2 M NaCl linear gradient. The final ITX purity, as assessed by densitometry of a 12% SDS/PAGE followed by Coomassie Blue staining, was >95%. Activity was tested using cytotoxicity test on Caco-2 cell monolayers [13].

### *In vitro* adhesion to Caco-2 cells assays

Washed bacteria were used to infect cell monolayers; 1 ml of a stationary phase culture of each strain was centrifuged at 13.000 g for 5 min and washed 3 times with sterile PBS. Pellet was resuspended in fresh MEM to get a final concentration of 1.5 x 10^7^ CFU of *C*. *perfringens*, which was added to cells monolayer to determine adhesive properties of each strain. Infected cells were incubated in a humidified atmosphere containing 5% CO_2_ at 37°C for 2 h. Cell monolayers were washed 3 times with sterile PBS to remove unattached bacteria. Then, Caco-2 cells were disrupted by distilled water immersion for 15 min and the amount of bounded bacteria was estimated by colony counting on neomycin blood agar plates (CFU/ml) after overnight incubation at 37°C under anaerobic conditions. Adherence was expressed as the percentage of attached bacteria to the cell monolayer relative to the total number of added bacteria. First, the effect of ITX on type E bacteria (strain CpE218) adherence was evaluated in Caco-2 cells treated with different concentrations of purified ITX for 2 h previous to infection with bacteria suspension. Changes in adherence of type E bacteria were quantified as the percentage of adherence relative to control cell monolayer. To test if ITX mediated changes in clostridia adherence are specific for type E bacteria, type E (CpE218) and type A (Cp88 and Cp31) bacterial adherence were evaluated after treat Caco-2 cells with 150 ng/ml Ia and 300 ng/ml Ib, changes in adherence were quantified as mentioned before.

### Neutralization of iota toxin induced changes in clostridia adherence

To test if ITX was responsible for the observed changes in clostridia adherence to Caco-2 cells, antibody neutralization experiments were performed. For these experiments anti-ITX IgY antibodies were obtained as previously described [30] using ITX antigen. Purified ITX (150 ng/ml Ia and 300 ng/ml Ib) solution was mixed with a solution containing ITX-neutralizing IgY antibody or negative control IgY. Mixtures were then incubated for 30 min at room temperature. Adherence assays were performed as described before using type E and type A strains on Caco-2 cells monolayers non-treated, treated with ITX or with ITX + antibodies, Changes in adherence were quantified as the percentage of adherence relative to control cell monolayer (non-treated).

### Scanning electron microscopy

After ITX treatment and *C*. *perfringens* infection, Caco-2 cell culture medium was replaced by ice-cold cacodylate buffer (0.1 M cacodylate, 0.01 M CaCl_2_, 0.01 M MgCl_2_, 0.09 M saccharose, pH 6.9) and prefixed by adding paraformaldehyde to a final concentration of 5%. After 10 min glutaraldehyde was added to a final concentration of 2%. Cells were dehydrated by incubation in a graded series of ethanol in 10% steps up to 100%, critical-point dried with liquid CO_2_ (EMS 850 Critical Point Drier de Electron Microscopy Sciences.) and covered with a thin palladium-gold (60–40%) film for conductivity by sputter coating (SC7620 Mini Sputer Coater de Thermo VG Scientific). Samples were examined in a field emission scanning electron microscope DSM982 Gemini at an acceleration voltage of 5 kV.

### Mice experiments

Conventionally reared male Swiss mice (20–25 g body weight) were used for *in-vivo* adhesion and displacement assays. Overnight cultures of *C*. *perfringens* type A (Cp88) and type E (Cp218) were prepared as mentioned before. Mice were fasted for 4 h with free access to water until 1 h before the beginning of the experiment. For adhesion assays, groups of four mice were inoculated intragastrically with 0.2 ml of *C*. *perfringens* cultures, containing approximately 1 x 10^7^ CFU of strain CpE218 or Cp88. Inoculated mice were monitored for the onset, duration and outcome of colonization by serial culture of fecal pellets in neomycin blood agar plates in anaerobic conditions at 37°C. For displacement assays, groups of four mice were inoculated intragastrically with 0.2 ml of *C*. *perfringens* cultures, containing approximately 1 x 10^6^ CFU of strain CpE218 and 1 x 10^7^ CFU of strain Cp88 (inhibitor/target ratio 1:10). Inoculated mice were monitored for the onset, duration and outcome of colonization by serial culture of fecal pellets in neomycin and nalidixic acid blood agar plates in anaerobic conditions at 37°C. At the end of the observation period, euthanasia was performed. Postmortem examinations were performed immediately after death on all the animals from each group. The gastrointestinal tract was collected and fixed by immersion in 10% formalin at pH 7.2 for a minimum of 24 h before being processed routinely to obtain 4 μm thick sections. Tissue sections were prepared and stained either with H&E or used for immunohistochemistry (IHC).

### Institutional Animal Care and Use Committee (IACUC) Approval

Studies presented here were reviewed and approved by the Comité Institucional para el cuidado y uso de animales de experimentación (IACUC) from the CICVyA-INTA, under protocols 32/2010 and 27/2011.

### Statistical analyses

Statistical analyses were performed using GraphPad Prism 5.0 software. Strain comparison at individual time points was performed by one-way ANOVA. To assess the overall trends of growth and the corresponding phenotypic traits over the time-course, area under curve (AUC) analysis was performed for each strain and strains were then compared by one-way ANOVA. Student’s t test was used when two groups of data had to be compared and Friedman test was used when more than two groups had to be compared. P values <0.05 were considered statistically significant.

## Results

### *C*. *perfringens* type A strains are inhibited by a type E soluble factor

**Fig 1:**
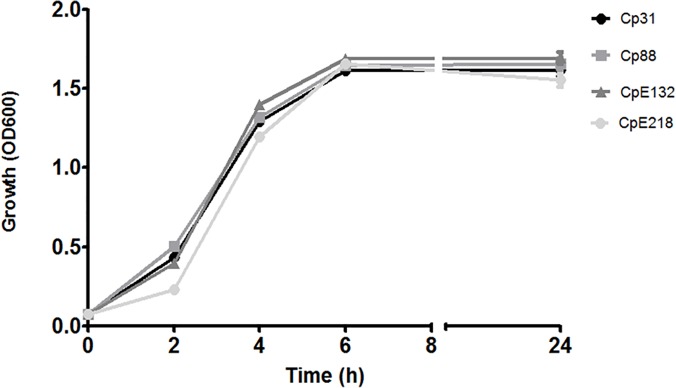
Growth curves of selected *C*. *perfringens* strains. The growth of two type E (CpE218 and CpE132) and two type A (Cp88 and Cp31) strains was measured by OD_600_ over 24 h. The patterns of growth were similar for all the strains. Results shown represent the average of three independent experiments; error bars indicate the standard error of the mean (SEM).

**Fig 2:**
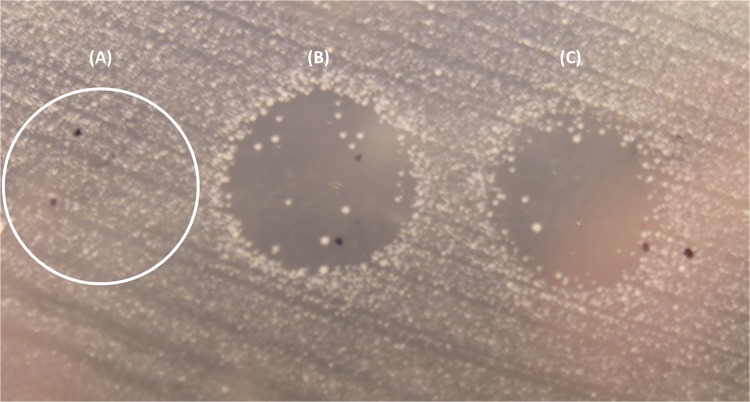
Result of the agar spot lawn diffusion assay. **(A)** A drop of *C*. *perfringens* type A strain (Cp31) supernatant was stabbed on a lawn of an indicator type A strain (Cp88) and partially through the agar beneath, no inhibition was observed. When type E supernatant was used, CpE218 and CpE132 (**B** and **C** respectively) a clear inhibition zone was observed.

Growth of selected strains (Table 1) was determined in order to rule out intrinsic growth differences between these strains that could interfere with results of subsequent experiments. Similar *in-vitro* growth rates (*P*>0.05) were calculated for these type A and type E strains (Fig. 1). Filter sterilized supernatants of type A and type E strains were tested for inhibitory activity. Both type E strains were able to inhibit tested type A strains (Fig. 2) but differences between inhibitory activities of type E strains were observed. CpE218 strain showed higher inhibitory effects than CpE132 and was also able to inhibit this last one (data not shown). None of the type A strains showed inhibitory activity against other type A or type E strains. Studies were made in an attempt to define the nature of the inhibitor factor. After treating type E supernatants 30 min at 80°C and 10 min at 100°C, inhibitory activity against *C*. *perfringens* type A strains (Cp31 and Cp88) was no longer present. A significant reduction in inhibitory activity was observed after treatment with trypsin (*P*<0.05) and proteinase K (*P*<0.05), while treatment of concentrated supernatants with papain did not reduce inhibitory activity (*P*>0.05). Heat inactivation and susceptibility to proteolitic enzymes suggest a proteinaceous nature of the detected inhibitory factor. Results are summarized on Table 2.

**Table 2:** Effects of enzymes and heat on type E antimicrobial activity.

**Treatment**	**Activity** ^a^ **(%)**
Control	100
Trypsin	35
Proteinase K	25
Papain	100
Temperature (time)	
4 (60 min)	100
24 (60 min)	100
37 (60 min)	100
42 (60 min)	100
60 (60 min)	100
80 (30 min)	0
100 (10 min)	0

^a^ Activity is the remaining antimicrobial activity expressed as percentage of the control.

### *C*. *perfringens* type E strains inhibit the growth of type A strains in co-culture

**Fig 3:**
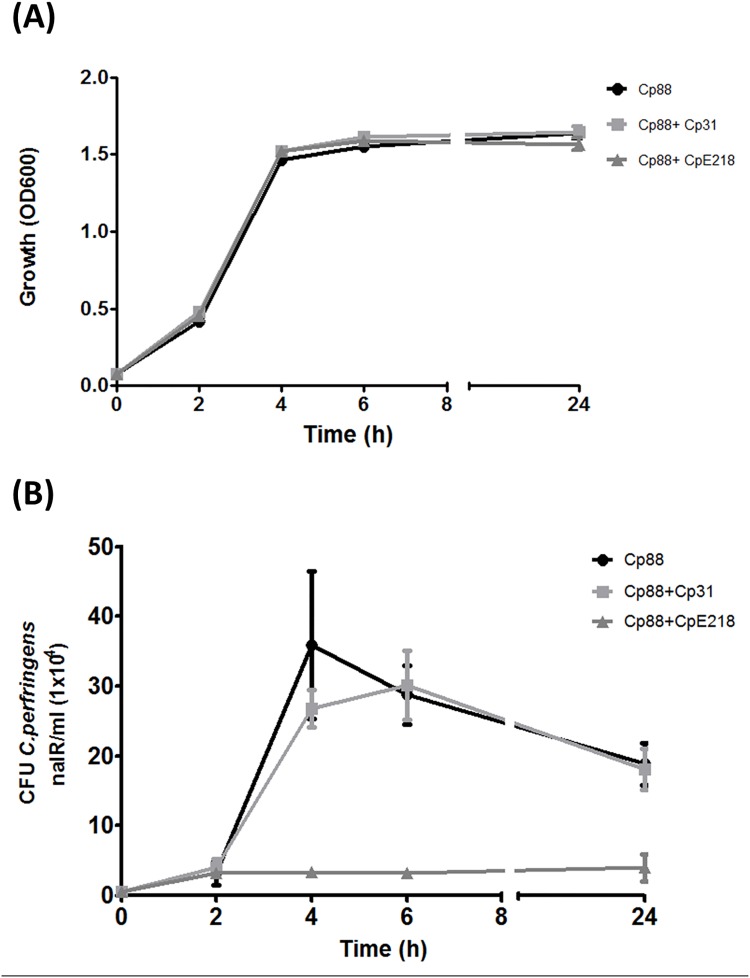
*C*. *perfringens* type E (CpE218) strain inhibits the growth of type A strain (Cp88). Both strains were co-cultured under anaerobic conditions. **(A)** Optical density at 600 nm shows no differences (*P>*0.05) in total clostridia cell density between monocultures (Cp88) and co-cultures with inhibitor type E strain (CP88 + CpE218) and non-inhibitor strain (Cp88 + Cp31). **(B)** Samples were analyzed for nalidixic acid resistant (nalR) CFU/ml every 2 h of culture. Compared to the expected number of nalidixic acid resistant Cp88 grown in monoculture, there was a significant reduction in the number of nalidixic acid resistant CFU counts since 4 h of culture onwards. No significant differences were observed when cultivated with a nalidixic acid sensitive non-inhibitor type A strain (Cp31). Results are the mean CFU counts obtained from three independent experiments; error bars indicate the standard deviation (SD).

When nalidixic acid resistant type A strain (Cp88) was co-cultured with nalidixic acid sensible type A or type E strains in BHI broth, no significant differences in optical density (OD600) were observed (P>0.05). These results suggest that total clostridia cell density was similar in monoculture and co-cultures (Fig. 3A). Experiments with nalidixic acid resistant type A and type E strains showed no significant differences between monoculture and co-cultures in counts of nalidixic acid resistant CFU after 2 h of anaerobic culture (*P*>0.05). In contrast, after 4 h of co-culture of type A and type E strains, counts of nalidixic acid resistant CFU were significantly lower when compared to monoculture of type A (Cp88) strain (*P*<0.001) (Fig. 3B). At 24 h, CFU count of nalidixic acid resistant type A strain (Cp88) in co-culture with type E strain was 6 times lower compared with monoculture (*P*<0.001). Co-culture with the nalidixic acid sensitive type A strain (Cp31), which did not show inhibitory effect over the Cp88 strain, produced a reduction in nalidixic acid resistant CFU count at 4 h, but these differences were not statistically significant (*P>*0.05). This may suggest that the effect of a non-specific mechanism like nutrient consumption or quorum sensing response. All the results are expressed in comparison with nalidixic acid resistant type A strain (Cp88) grown in monoculture. Multiplex PCR assays probed that nalidixic acid resistant colonies were only type A and confirmed the specificity of the selective plating protocol.

### Type E clostridia reduce type A cell adhesion

**Fig 4:**
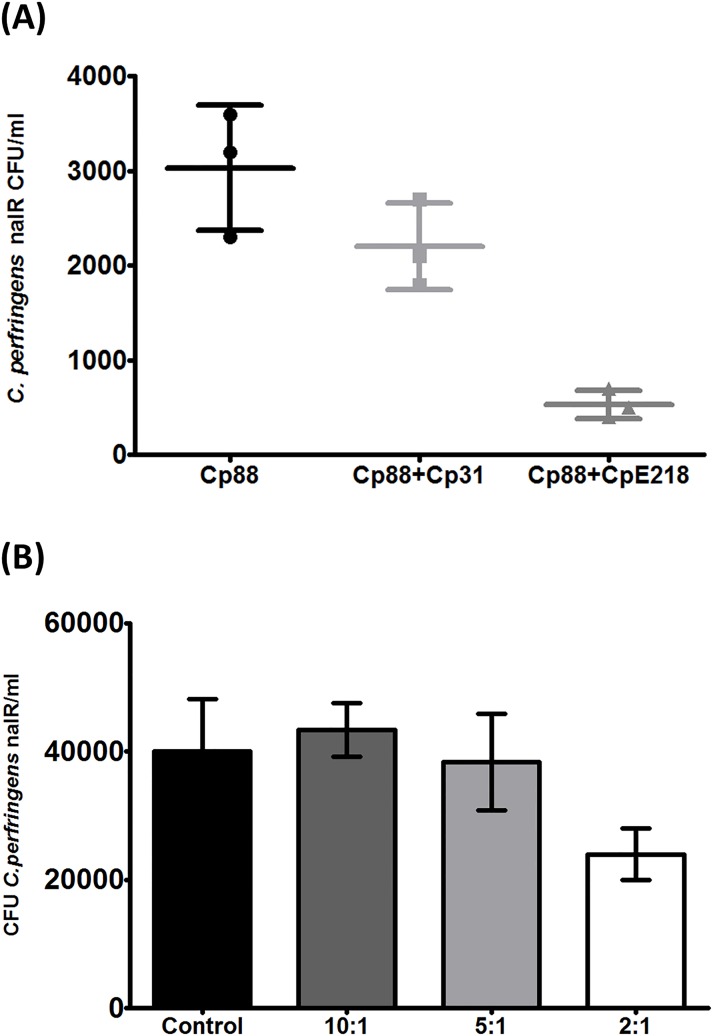
Type E clostridia reduce type A cell adhesion. **(A)** *C*. *perfringens* type E (CpE218) strain reduces adherence of type A strain (Cp88) to Caco-2 cells. Caco-2 cells were incubated with DMEM medium containing bacteria. Cp88: Nalidixic acid resistant type A strain alone; Cp88+Cp31: Mix of nalidixic acid resistant type A strain with a non-inhibitor type A strain but sensitive to nalidixic acid; Cp88+CpE218: Mix of nalidixic resistant type A strain with a type E strain. As mentioned, both strains were added simultaneously. Adhesion of Cp88 alone was considered as a control for the adhesion assay. **(B)** Type E cells displace type A cells attached to Caco-2 cells only at higher type A/type E ratio. The number below each column represents the ratio between attached type A and free competing type E cells. Results are the mean nalidixic acid resistant (nalR) CFU counts from three independent experiments; error bars indicate the standard deviation (SD).

Competitive inhibition and displacement between both strains were examined in adhesion assays on Caco-2 cells monolayers. When both strains were added at the same time, a reduction in attached type A bacteria was observed as determined by nalidixic acid resistant CFU counts compared with controls (*P<* 0.0001) (Fig. 4A). The addition of type A Cp31 strain without inhibitory effect, caused a slight reduction in nalidixic acid resistant CFU counts which was *not statistically significant* (P>0.05). In displacement assays (Fig. 4B), type A clostridia were first allowed to attach to Caco-2 cells for 2 h before the addition of type E clostridia. These displacement assays showed that type E is not able to displace significantly (*P>*0.05) the cell-attached type A clostridia. After increasing the relative number of type E cells (inhibitor/target ratio 1:2) it was possible to observe a significant decrease in nalidixic acid resistant CFU counts (*P<*0.05). The addition of another type A strain (Cp31) without inhibitory effect, did not show significant effect on displacement assays in any of the tested ratios (P>0.05). Multiplex PCR assays confirmed that nalidixic acid resistant colonies were only type A and confirmed the specificity of the selective plating protocol.

### Iota toxin increases type E adherence while decreases type A adherence

**Fig 5:**
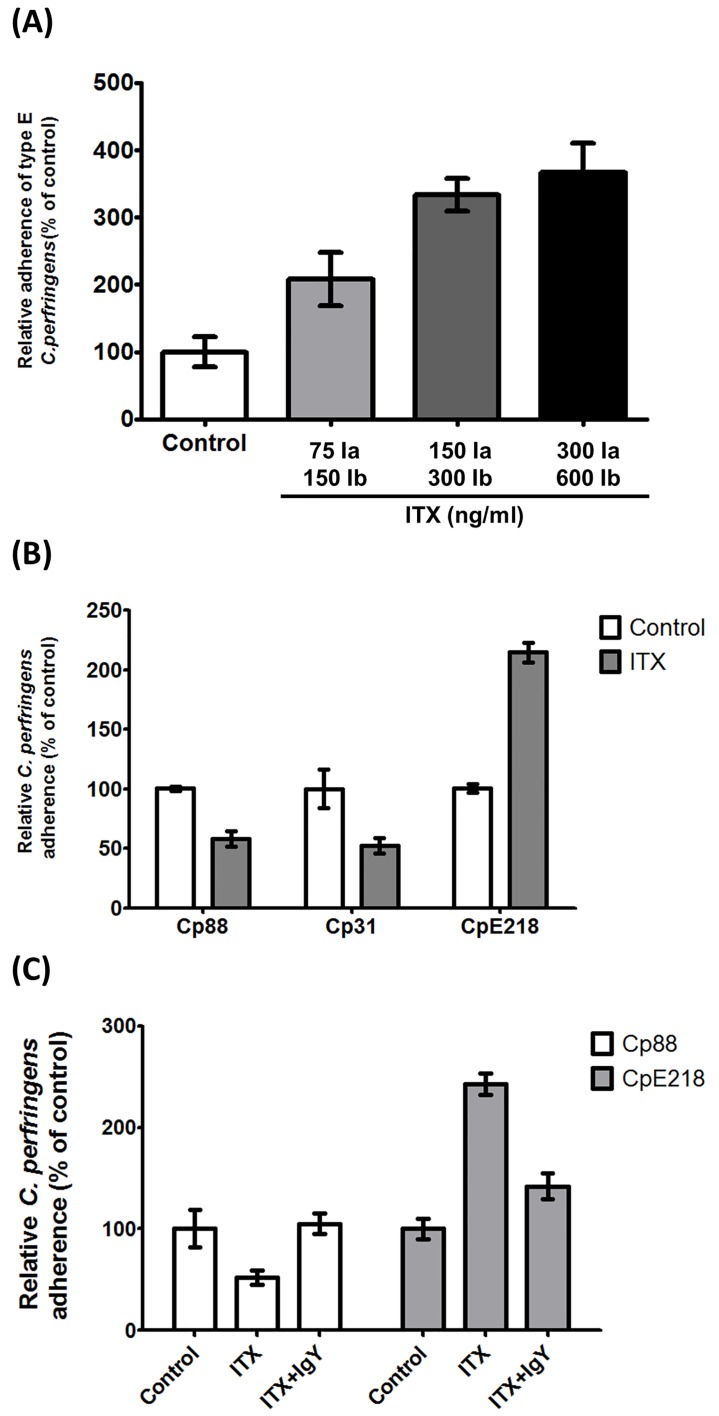
Iota toxin increases type E adherence while decreases type A adherence. **(A)** ITX treatment increases the adherence of *C*. *perfringens* type E (CpE218). Cells were incubated with the indicated toxin concentrations. 75ng/ml Ia +150ng/ml Ib, 150ng/ml Ia +300ng/ml Ib and 300ng/ml Ia +600ng/ml Ib. After ITX treatment, cells monolayers were incubated with type E bacteria. Cells were lysed and total clostridia were plated onto neomycin blood agar plates for counting. **(B)** ITX treatment increases the adherence of type E bacteria (CpE218), and reduce adherence of type A bacteria (Cp88—Cp31). Cells were treated with 150 ng/ml Ia and 300 ng/ml Ib (ITX), and washed 3 times with PBS. After ITX treatment, cells monolayers were incubated with the indicated strains. **(C)** ITX induced changes in bacterial adherence were neutralized by addition of anti-ITX IgY. Cells were treated with 150 ng/ml Ia and 300 ng/ml Ib (ITX) with or without neutralizing IgY. After treatment, cells monolayers were incubated with the indicated strains. Attachment levels were quantified as percent of bacterial adherence relative to untreated cells. Attachment levels were quantified as percent of bacterial adherence relative to untreated cells. Results shown represent the average of three independent experiments; error bars indicate the standard error of the mean (SEM).

**Fig 6:**
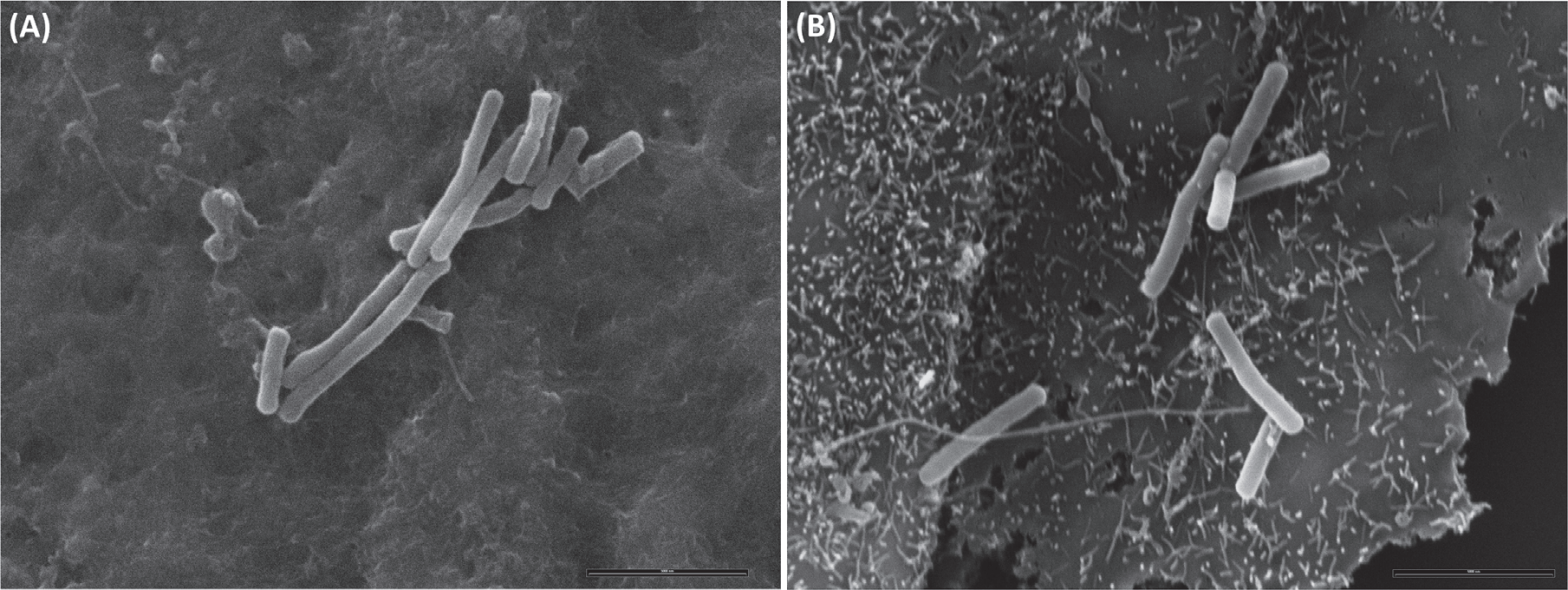
Scanning electron microscopy of *C*. *perfringens* interacting with epithelial cells. Caco-2 cells were incubated for 2 h with PBS buffer **(A)** or ITX **(B)**. No superficial protrusions were observed in PBS treated cells, although bacteria were attached to the cells surface after 2 h of anaerobic incubation. ITX treatment induced the formation of membrane protrusions in Caco-2 cells and clostridia cells were adhered to these cellular deformations.

Since previous reports have shown that ITX increases adhesion of bacteria to eukaryotic cells [19], differences in adherence between type E and type A strains and the influence of ITX were analyzed. The average percentage of adherence of C. perfringens type A and type E were 76.3% and 35.5% respectively, which suggest that type A strains bind more effectively to Caco-2 cells than type E strains. However, treatment of monolayers with ITX increased the number of type E bacteria attached to cells in a dose dependent manner (Fig. 5A). Comparative adhesion assay between type E and type A strains, shows about a 2 fold increase in enterocyte-bounded type E clostridia after treatment with ITX, as compared to untreated controls (*P<*0.0001) (Fig. 5B). A 58.4% and 52% reduction was observed in bacterial adhesion values for type A strains Cp88 and Cp31 as compared with controls (*P<*0.001). To definitively evaluate whether observed adherence changes were ITX-mediated neutralization experiments were performed. Preincubation of ITX with IgY anti-ITX neutralized the changes of type A and type E strains adherence on Caco-2 cells (*P<*0.05) (Fig. 5C). A control of IgY obtained from non-immunized animals was not able to avoid the observed effects (data not shown). Scanning electron microscopy studies revealed that attached bacteria were not homogeneously distributed but forming small aggregates on both treated and control monolayers (Fig. 6), which may hinder bacterial counts. However, type E bacteria attached to surface protrusions were observed in ITX treated monolayers. These protrusions were not observed in these control cells, although some few bacteria were attached to cells surface of non-treated cells.

### *C*. *perfringens* type E bacteria attach to small intestinal mucosa

**Fig 7:**
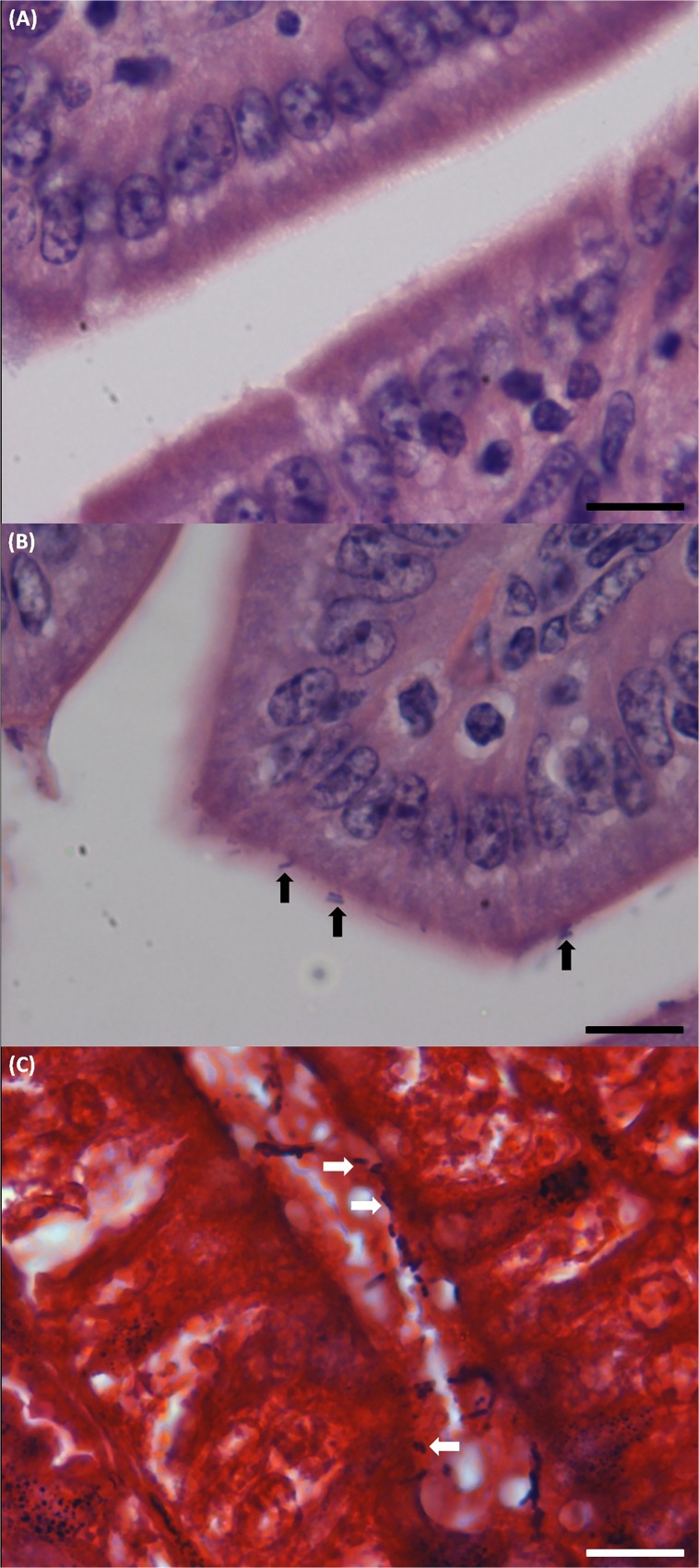
Histological analysis of sample sections of ileum of the *C*. *perfringens* challenged mice. Mice were intragastrically inoculated with the indicated strains and then sacrificed 48 h post-infection. **(A)** Hematoxylin and eosin staining of sample sections of ileum of the mice challenged with type A strain (Cp88), no *C*. *perfringens* compatible attached bacteria were observed. Scale bar, 20 μm. **(B)** In type E challenged mice many rod shaped bacteria compatible with *C*. *perfringens* (black arrows) were observed intimately attached to the intestinal mucosa. Scale bar, 20 μm. **(C)** Gram staining of a sample section of ileum of one of the type E challenged mice: Gram positive rod shaped bacteria compatible with *C*. *perfringens* attached to the gut mucosa (white arrows). Scale bar, 20 μm.

**Fig 8:**
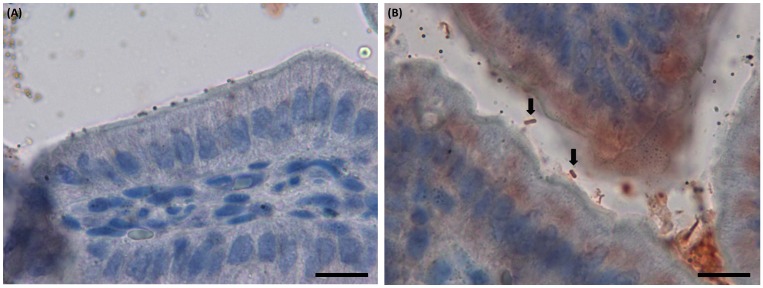
Immunohistochemical analysis of sample sections of ileum of the *C*. *perfringens* challenged mice. Mice were intragastrically inoculated with the indicated strains and then sacrificed 48 h post-infection. **(A)** Sample sections of ileum of the mice challenged with type A strain (Cp88), no attached *C*. *perfringens* were observed. Scale bar, 20 μm. **(B)** In type E challenged mice, many red bacilli corresponding with *C*. *perfringens* (black arrows) were observed intimately attached to the intestinal mucosa. Scale bar, 20 μm.

In order to evaluate if the adherence of *C*. *perfringens* type E to Caco-2 cells is also reproduced in epithelial cells *in vivo*, mice were infected with both toxinotypes. After challenge with type E (CpE218) and type A (Cp88) strains, none of the challenged mice developed clinical signs during the examination period. Onset of *C*. *perfringens* colonization was determined by periodic fecal cultures. Colonization onset after challenge with strains CpE218 or Cp88 was similar in both groups. Excretion of *C*. *perfringens* was detected in 60% of the inoculated animals during the first 24h. After two consecutive negative fecal cultures (48 h post challenge), mice were sacrificed. None of the animals exhibited gross lesions and no histological changes were observed in any of the challenged animals. Despite this, histological analysis of jejunum and ileum sections of type E challenged mice revealed many Gram positive rod shaped bacteria (Fig. 7B-C) and IHC confirmed the identity of the bacteria as *C*. *perfringens* (Fig. 8B). In type A challenged mice no attached bacteria compatible with *C*. *perfringens* were observed (Fig. 7A; Fig. 8A).

### Type E bacteria reduce intestinal colonization by type A strains *in-vivo*

Inoculation of mice with CpE218 and Cp88 strains resulted in colonization of 60% of the mice. Extension of colonization was variable, ranging from 6 to 24 h after challenge. Mice intestinal colonization rate and colonization extension was similar for type E and type A strains (*P>*0.05). None of the mouse showed signs of disease or died during the observation period after the challenge. All negative control mice remained negative for *C*. *perfringens* bacteriology detection and showed no signs of disease throughout the observation period. When mice were challenged with a mix of type A and type E bacteria (10:1 ratio), type A colonization rate showed a significant reduction (30% vs. 60%). Also, it was possible to observe a reduction in the duration of intestinal colonization by type A clostridia (18 h vs. 2 h; *P<*0.005).

## Discussion

*C*. *perfringens* type E has been identified as a pathogen associated with hemorrhagic enteritis and sudden death in ruminants [6, 7, 10]. Although numerous studies describe the mechanism involved in cell intoxication by iota toxin produced by *C*. *perfringens* type E strains, little is known about the pathogenesis of this toxinotype. Previous studies report a clonal relationship between different type E isolates obtained from a single diseased bovine and between isolates from diverse geographical locations, suggesting a clonal predominance and successful expansion of one type E clone within the bovine gut ecological niche [7, 23]. The results of the present study suggest that modifications on the cellular surface induced by iota toxin and the ability to inhibit the growth of competitor *C*. *perfringens* strains could be at least some of the mechanisms implied in the initials stages of gut invasion by type E bacteria and the development of clonal sub-populations.

Bacterial species that comprise highly diverse communities, as the intestinal microbiota, are often engaged in a fierce arms race over resources and space. The importance of intra-specific inhibition as a pathogenic strategy for some *C*. *perfringens* has been postulated for strains producing necrotic enteritis in poultry since clonal dominance of pathogenic over commensal strains has been observed [24, 31]. The results of the present report show that the observed intra-species inter-strain growth inhibition caused by type E *C*. *perfringens* is mediated by a bacteriocin-like substance secreted by the bacterium into its microenvironment. Heat inactivation and susceptibility to proteolitic enzymes suggest a proteinaceous nature of this inhibitory factor, although further characterization is needed. It is known that all the *C*. *perfringens* toxinotypes are able to produce bacteriocins that can affect other *C*. *perfringens* strains [32]. Timbermont et al. [24] and Watson et al. [33] reported that most strains implicated in poultry necrotic enteritis (46%) and human food poisoning (79%) outbreaks respectably produced some kind of bacteriocins; however, only 15% of isolates from healthy chickens and 18% from healthy persons produced bacteriocins, suggesting a relationship between bacteriocin production and the ability to cause intestinal disease in humans and animals.

Bacteriocins are substances commonly involved in intra-species growth inhibition [25]. The dominant reason for bacteria to produce bacteriocins is to provide the producing organism with an ecological advantage over its most likely competitors [34]. Bacteriocins are proteinaceous toxins that facilitate their producing organisms to defend their habitat against invaders, limit the advance of neighbouring cells [35] or invade an established bacterial community [36, 37]. Therefore, the production of bacteriocins by *C*. *perfringens* type E (and probably others toxinotypes) could be important to improve their survival within the intestinal environment either defending their space from other strains or invading an established environment. Our *in- vitro* experiments showed that only a high proportion of *C*. *perfringens* type E could displace established type A cells, suggesting a defensive role for bacteriocins in the intestinal environment for type E strains.

Similar to *C*. *perfringens* type E and the iota toxin, *C*. *spiroforme* causes diarrheic deaths that are spontaneous or antibiotic induced in rabbits [11] and perhaps in humans [38], via a binary iota-like toxin referred to as CST. Closely related to the iota toxin and CST is the iota-like toxin produced by *C*. *difficile* (CDT) [18]. The protein components of these toxins are interchangeable, thus generating biologically active chimeras that demonstrate conserved functionality among these clostridial species [39]. Interestingly *C*. *difficile*, *C*. *perfringens*, and *C*. *spiroforme* are all associated with gastrointestinal diseases in humans as well as in animals [1, 2] and the synthesis of common binary toxins with interchangeable protein components probably reveals a shared evolutionary path for these ubiquitous pathogens in a common niche. The actin-ADP-ribosylating binary toxins of clostridia, not only affect the actin cytoskeleton and induce the formation of microtubule based extensions, but induce a vesicle rerouting with the consequent secretion of extracellular matrix proteins like fibronectin to increase bacterial adherence [19, 20]. We have observed similar changes at the enterocyte surface of cultured cells induced after iota toxin incubation that resulted in an increased adherence of *C*. *perfringens* type E bacteria. However, we found that this cellular alteration could be a disadvantage for non-producing iota toxin strains, suggesting that type E strains codify not only for iota-toxin but specific factors of adherence to this meshwork of microtubule based structures and extracellular matrix proteins.

In order to understand how type E strains could deal with type A strains in the intestinal environment, mice were first inoculated with both strains separately. Colonization was observed in mice by either *C*. *perfringens* type A or type E inoculation. A comparable pattern of *C*. *perfringens* type E adherence to cultured epithelial cells altered by iota toxin was also detected in the epithelium of the gastrointestinal tract of mice challenged with this toxinotype but no with others. Also, we detected a comparable distribution and sort of adherence of *C*. *perfringens* in the gastrointestinal tract of bovines with natural *C*. *perfringens* type E disease [7], suggesting that type E also induced these epithelial alteration and attachment in ruminants gut. In mice inoculated with type A and a minor fraction (10:1) of type E clostridia, type colonization was significantly reduced. The experiments with mice suggest that type E has the ability to inhibit other *C*. *perfringens* strains *in-vivo* and it would be an explanation for previous observation of clonal predominance [7, 23] and intra-specific inhibition of this toxinotype. Short times of fecal recovery of both strains may suggest an apparent negative competitive interaction between both strains. In well-mixed, unstructured environments (as the intestinal lumen) producers of weak bacteriocin-like activity are selectively advantageous and overcome producers of strong bacteriocin-like [40]. From our *in-vitro* results, we assume that type E produces a strong bacteriocin-like activity against the selected sensitive type A strain but this type A strain did not show any inhibitory activity against type E. A possible explanation for the mice result is that the strong inhibitory activity of type E strain eliminated type A cells but the relatively low proportion of type E cells was not able to overcome the rough intestinal environment and to multiply enough to be recovered by regular anaerobic culturing procedures. However, *C*. *perfringens* cells were observed attached to altered enterocytes in the small intestine of mice either if type E was or not co-administered with the sensitive type A strain. In contrast, attachment was not observed in animals inoculated only with several type A strains obtained from apparently healthy animals.

The ability of type E strains to colonize and create a structured environment seems to be important for the success of this toxinotype and constitutive strong bacteriocin production was probably acquired after this sessile lifestyle, protecting their space from invaders and improving the fitness of determined type E clones. Bacteriocinogenic bacteria are supposed to be rare at the time of their origin and they cannot increase from low frequencies in an unstructured habitat, consequently structured habitats are necessary for the evolution of bacteriocin [40]. Therefore, it could be hypothesized that bacteriocin production exists because of iota toxin which allows the attachment of type E strains to enterocytes, a structured habitat.

The specialized adhesive properties of several species of *Clostridium* to enterocytes and the synthesis of inhibitory substances seem most likely to be an adaptive response to a variety of factors present in a complex and competitive medium like the gut environment. These characteristics would facilitate the development of clonal populations, observed in natural cases of enteritis due to *C*. *perfringens* type E [7, 23]. However, it remains to be elucidated the connection between initial attachment with the damage observed in sick animals, particularly if iota toxin only facilitates the bacteria attachment or it is also responsible for the enteritis and associated epithelial damage observed in natural cases of type E disease. Further work is necessary to define the predisposing factor that would increase the damage produced after *C*. *perfringens* type E colonization in bovine intestine.

## Supporting Information

S1 Checklist ARRIVE Checklist. (DOCX)
