# Supplementary material for: Clostridium perfringens Type E Virulence Traits Involved in Gut Colonization
Source: PLoS One. 2015 Mar 23;10(3):e0121305. doi: 10.1371/journal.pone.0121305 (PMC4370460; doi:10.1371/journal.pone.0121305)

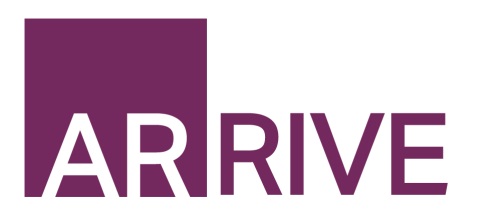


The ARRIVE Guidelines Checklist

Animal Research: Reporting In Vivo Experiments

Carol Kilkenny^1^, William J Browne^2^, Innes C Cuthill^3^, Michael Emerson^4^ and Douglas G Altman^5^

*^1^The National Centre for the Replacement, Refinement and Reduction of Animals in Research, London, UK, ^2^School of Veterinary Science, University of Bristol, Bristol, UK, ^3^School of Biological Sciences, University of Bristol, Bristol, UK, ^4^National Heart and Lung Institute, Imperial College London, UK, ^5^Centre for Statistics in Medicine, University of Oxford, Oxford, UK.*

|  | | ITEM | RECOMMENDATION | Section/ Paragraph |
| --- | --- | --- | --- | --- |
| 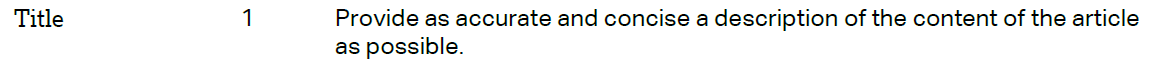 | | | Title. |  |
| 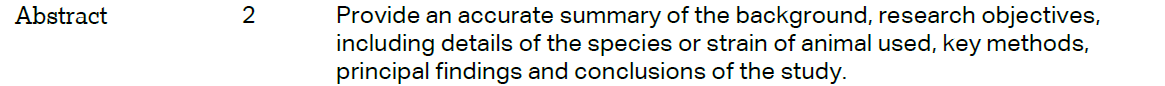 | | | Abstract. |  |
| INTRODUCTION | | |  |  |
| 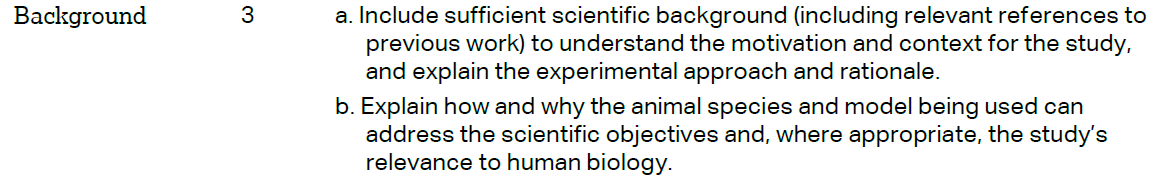 | | | Introduction/ 1^st^, 2^nd^ paragraph. Introduction/3^rd^ paragraph. |  |
| 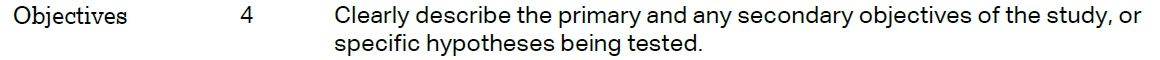 | | | Introduction/ 4^th^ paragraph. |  |
| METHODS | | |  |  |
| 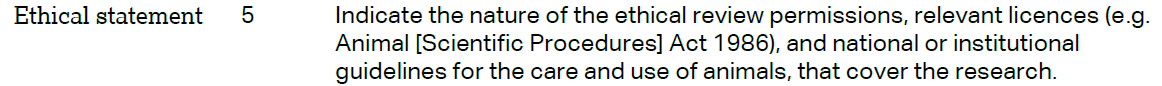 | | | Materials and methods/ Institutional Animal Care and Use Committee (IACUC) Approval |  |
| 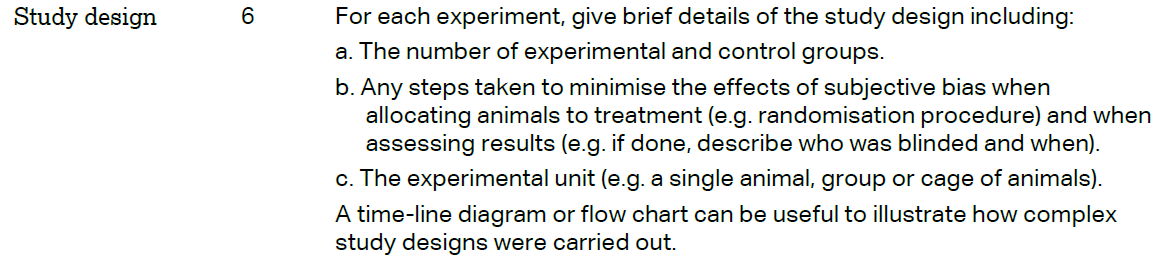 | | | Materials and methods/Mice experiments |  |
| 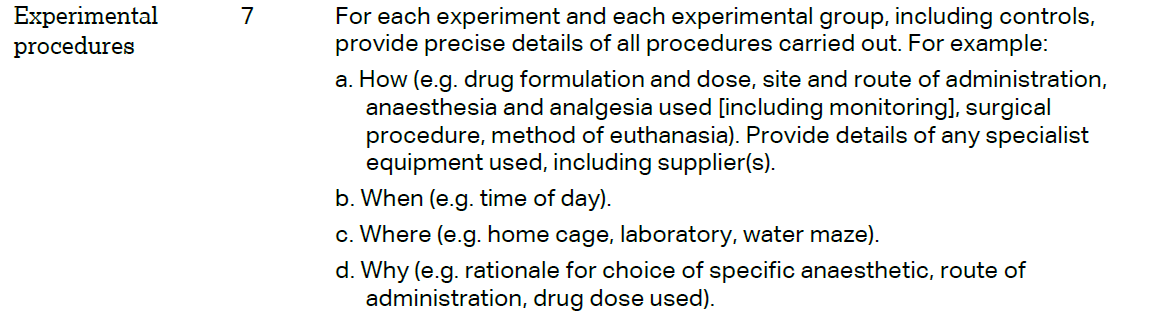 | | | Materials and methods/Mice experiments |  |
| 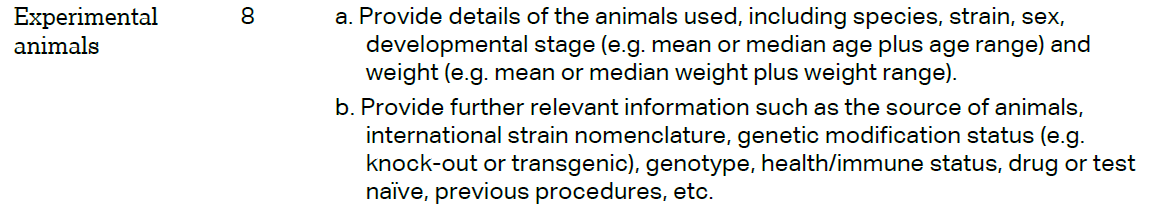 | | | Materials and methods/Mice experiments |  |

The ARRIVE guidelines. Originally published in *PLoS Biology*, June 2010^1^

| 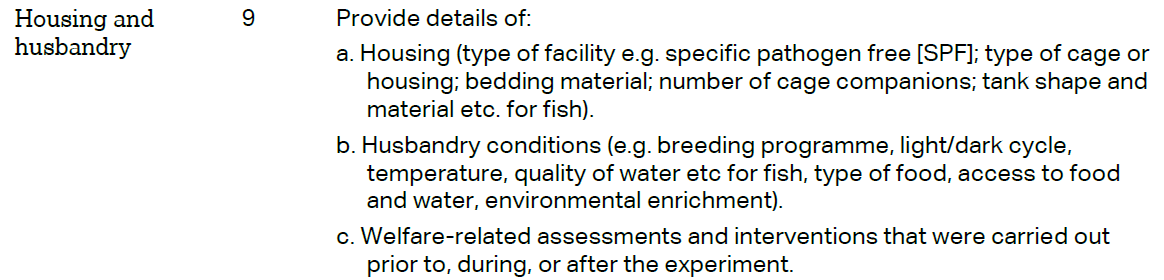 | Materials and methods/Mice experiments | |
| --- | --- | --- |
| 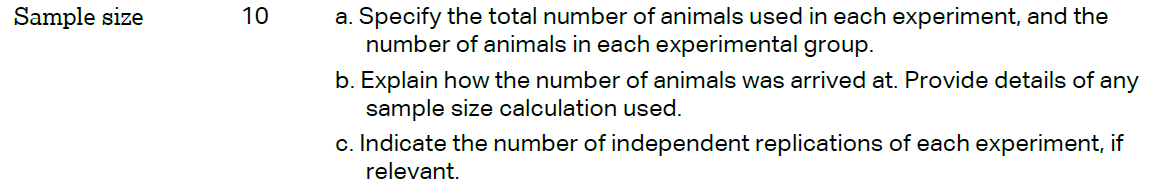 | Materials and methods/Mice experiments | |
| 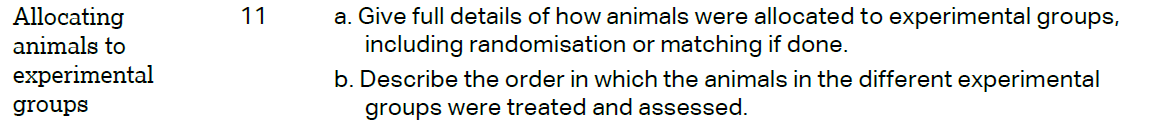 | Materials and methods/Mice experiments | |
| 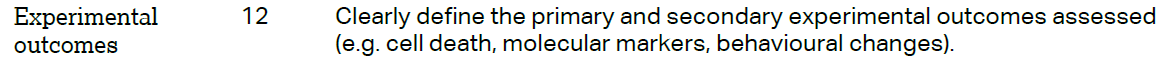 | Materials and methods/Mice experiments | |
| 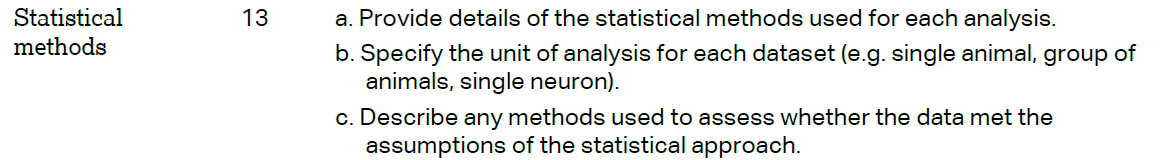 | Materials and methods/Statistical analyses | |
| RESULTS |  | |
| 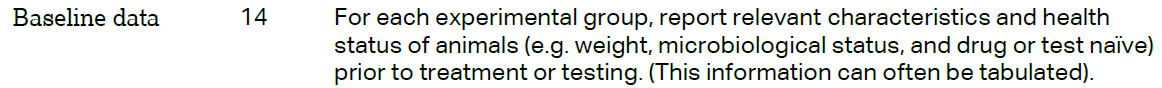 | Results/ Type E bacteria reduce intestinal colonization by type A strains *in-vivo*. | |
| 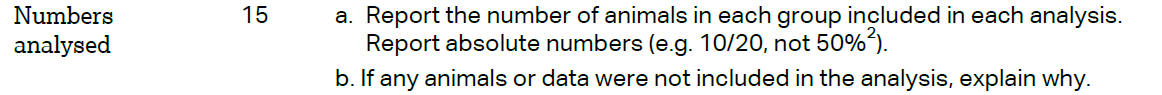 | Results/ Type E bacteria reduce intestinal colonization by type A strains *in-vivo*. | |
| 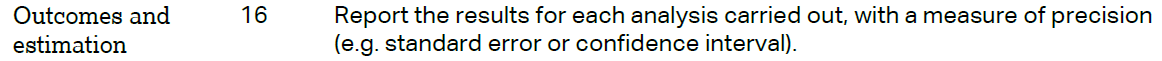 | Results/ Type E bacteria reduce intestinal colonization by type A strains *in-vivo*. | |
| 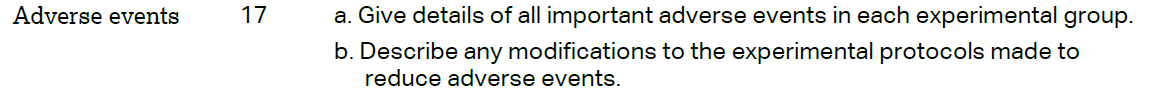 | Results/ Type E bacteria reduce intestinal colonization by type A strains *in-vivo*. | |
| DISCUSSION |  | |
| 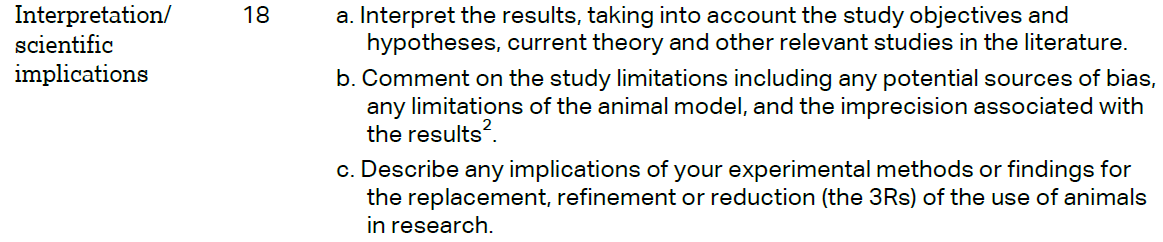 | Discussion/1^st^, 2^nd^, 3^rd^ paragraph.  Discussion/4^th^, 5^th^ paragraph.  Discussion/4^th^ paragraph. | |
| 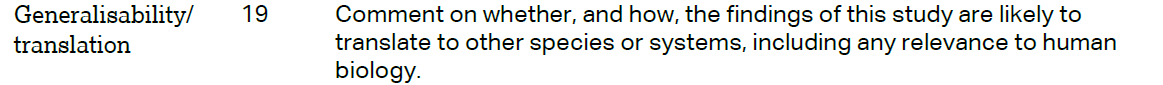 | Discussion/ 2^nd^, 3^rd^ paragraph. | |
| 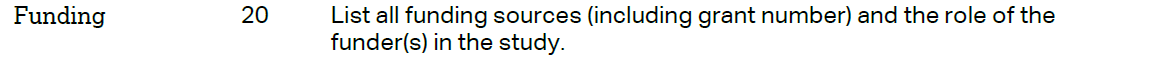 | | Acknowledgements |


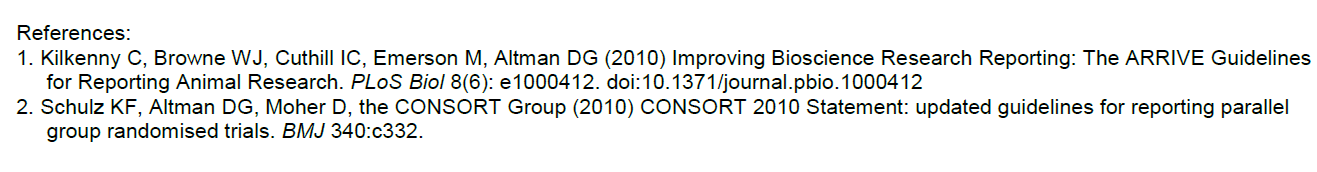

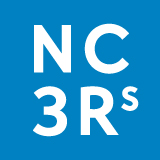

Supplement: S1 Checklist — (DOCX) [file pone.0121305.s001.docx]
